# Predictors of micronutrient deficiency among children aged 6–23 months in Ethiopia: a machine learning approach

**DOI:** 10.3389/fnut.2023.1277048

**Published:** 2024-01-05

**Authors:** Leykun Getaneh Gebeye, Eskezeia Yihunie Dessie, Jemal Ayalew Yimam

**Affiliations:** ^1^Department of Statistics, College of Natural Science, Wollo University, Dessie, Ethiopia; ^2^Department of Pediatrics, Cincinnati Children’s Hospital Medical Center, University of Cincinnati, College of Medicine, Cincinnati, OH, United States

**Keywords:** machine learning, child micronutrient deficiency, AUROC, spatial variation, Ethiopia

## Abstract

**Introduction:**

Micronutrient (MN) deficiencies are a major public health problem in developing countries including Ethiopia, leading to childhood morbidity and mortality. Effective implementation of programs aimed at reducing MN deficiencies requires an understanding of the important drivers of suboptimal MN intake. Therefore, this study aimed to identify important predictors of MN deficiency among children aged 6–23 months in Ethiopia using machine learning algorithms.

**Methods:**

This study employed data from the 2019 Ethiopia Mini Demographic and Health Survey (2019 EMDHS) and included a sample of 1,455 children aged 6–23 months for analysis. Machine Learning (ML) methods including, Support Vector Machine (SVM), Logistic Regression (LR), Random Forest (RF), Neural Network (NN), and Naïve Bayes (NB) were used to prioritize risk factors for MN deficiency prediction. Performance metrics including accuracy, sensitivity, specificity, and Area Under the Receiver Operating Characteristic (AUROC) curves were used to evaluate model prediction performance.

**Results:**

The prediction performance of the RF model was the best performing ML model in predicting child MN deficiency, with an AUROC of 80.01% and accuracy of 72.41% in the test data. The RF algorithm identified the eastern region of Ethiopia, poorest wealth index, no maternal education, lack of media exposure, home delivery, and younger child age as the top prioritized risk factors in their order of importance for MN deficiency prediction.

**Conclusion:**

The RF algorithm outperformed other ML algorithms in predicting child MN deficiency in Ethiopia. Based on the findings of this study, improving women’s education, increasing exposure to mass media, introducing MN-rich foods in early childhood, enhancing access to health services, and targeted intervention in the eastern region are strongly recommended to significantly reduce child MN deficiency.

## 1 Introduction

Micronutrient (MN) deficiencies are a major public health problem around the world, contributing to childhood morbidity and mortality. The burden of this problem is disproportionately high in low- and middle-income countries, particularly in Sub-Saharan Africa, including Ethiopia (1, 2). MN deficiencies mainly occur when people lack access to MN-rich foods like fruits, vegetables, animal products, and fortified foods. MN deficiencies lower immune capabilities and increase the overall risk of infection-related mortality, particularly diarrhea, measles, malaria, and pneumonia, which are among the world’s top ten leading causes of death (1, 3). MNs are only minimally required; however, their lack in the diet has a severe impact on the survival and development of children. Furthermore, MN deficiency contributes to stunting, wasting, weakened immunity, and delays in cognitive development (1, 3, 4).

Vitamin A (VA) and Iron are essential micronutrients that are crucial for the growth and development of children and their deficiency causes significant public health problem in children (5). Iron deficiency is a primary cause of anemia and has serious health consequences for both women and children. VA plays an important role in maintaining the epithelial tissue in the body. Its severe deficiency causes eye damage and is the leading cause of preventable childhood blindness. Moreover, VA deficiency increases the severity of infections such as measles and diarrheal disease in children and slows recovery from illness. It is common in dry environments where fresh fruits and vegetables are not readily available (3).

According to the 2019 United Nations Children’s Fund report, 340 million children globally suffered from hidden hunger as a result of MN deficiency (6). In Africa, less than one-third and one-half of children aged between 6 and 23 months met the minimum criteria for dietary diversity and meal frequency, respectively. According to the 2019 Ethiopian Mini Demographic Health Survey (EMDHS) report, the consumption of foods rich in VA and iron, which are the major MN deficiency indicators, remains low among young children in Ethiopia. Thirty-nine percent of children aged 6–23 months consumed foods rich in VA during the 24 h before the interview, whereas 24% consumed iron-rich foods (3).

Empirical studies have identified several factors associated with insufficient minimum dietary diversity, including limited access to media such as newspapers, magazines, and radio; lower education level of fathers; fewer antenatal care visits; younger child age; working in agriculture, and poorest household wealth index (1, 4, 6–8). However, the typical logistic and multilevel models employed in these studies were unable to identify the most important predictors. Identifying predictors of MN deficiency and taking corrective action are critical in reducing MN deficiency. Prioritizing predictors based on their contribution in predicting MN deficiency will be cost effective and simple to implement but has not yet been considered. Machine learning (ML) algorithms, which intersects statistical learning and artificial intelligence research, are used to explore large amounts of data to discover unknown patterns or relationships and show the share of predictors for a particular problem (9, 10). In addition, ML helps to develop predictive models and the selection of the most important predictors.

Hence, the ML algorithm is the ideal candidate statistical model for addressing these statistical modeling issues. These models have demonstrated high performance in solving classification problems compared to the conventional statistical models applied to select the most important predictors. The availability of diverse alternative models to be selected as the best fit for a predictive model is one of the most important features behind the use of ML algorithms. Among others, the five widely used ML models considered in this study are Support Vector Machine (SVM), Logistic Regression (LR), Neural Network (NN), Random Forest (RF), and Naïve Bayes (NB) (9–14).

The most significant predictors of MN deficiency were determined after evaluating these multiple models and choosing the model that best fit the data under consideration in this study. This enables health professionals, policy designers and implementers, and interventions geared towards addressing challenges posed by MN deficiency to concentrate their efforts on the most reliable predictors and take corrective actions. To the best of our knowledge, no previous study has used ML modeling to determine the factors that predict MN deficiency in Ethiopia and other East African nations. The main objective of this study was to identify the most important predictors of childhood MN deficiency in Ethiopia by evaluating various ML algorithms that most accurately and efficiently predict micronutrient deficiency.

## 2 Materials and methods

### 2.1 Data source and sampling procedure

This analysis involved the Ethiopia Mini Demographic and Health Survey (EMDHS), which was collected through a nationally representative, cross-sectional, and household-based survey conducted in Ethiopia in 2019. The data collection used a two-stage cluster sampling design with stratification into urban and rural regions. Twenty-one sampling strata were obtained after stratifying each region into urban and rural areas. In the first stage, 305 Enumeration Areas (EAs) (93 urban EAs and 212 rural EAs) were chosen with a probability proportional to the EA size in each stratum. In the second stage, 30 households were randomly selected from each EA using an equal probability method from the fresh list of households, resulting in a total of 8,663 households with 1,463 children aged 6–23 months (3).

### 2.2 Study variables and measurements

#### 2.2.1 Outcome variable

The outcome variable in this study was the MN deficiency status of children aged 6–23 months, which was derived based on the MN intake status from respondents’ report. It was mainly computed from the VA and Iron rich foods consumed in the last 24 h prior to the data collection among children aged 6–23 months. We classified children’s MN deficiency status into two groups: “Yes” outcomes if the respondent reported that the child did not consume any of the minimum recommended MNs, and “No” outcomes if the child had consumed at least one of the minimum recommended MNs (1).

A child was grouped in the MN deficient category in VA if he or she had not consumed any of the seven VA-rich foods in the 24 h prior to the data collection. The seven VA rich foods include: i. eggs; ii. meat (beef, hog, lamb, or chicken); iii. Pumpkin, carrots, and squash; iv. any dark green leafy vegetables; v. mangoes, papayas, and other fruits containing VA; vi. liver, heart, and other organs; and vii. Fish or shellfish. Similarly, a child was deemed MN deficient in Iron if she or he did not eat anything from the four food groups that were high in Iron: eggs, meat (beef, hog, lamb, or chicken), liver, heart, and other organs, fish, or shellfish. Hence, in this study, the MN deficiency status of the child was determined as MN deficient if the child was MN deficient in both groups (VA and Iron) and labeled “Yes” and “No” otherwise. The outcome variable is MN deficiency (Y), which is defined for an individual child as:


$$y_{i}=\{\begin{array}{c} 1,\mathrm{if}\;a\;\mathrm{child}\;i\;\mathrm{had}\;\mathrm{received}\;\mathrm{none}\;\mathrm{of}\;\mathrm{the}\;\mathrm{minimum}\;\mathrm{recomended}\;MNs\;\\ \;0,\mathrm{if}\;a\;\mathrm{child}\;i\;\mathrm{had}\;\mathrm{eaten}\;\mathrm{atleast}\;\mathrm{one}\;\mathrm{of}\;\mathrm{the}\;\mathrm{minimum}\;\mathrm{recomended}\;MNs\; \end{array}$$

#### 2.2.2 Predictors in the model

The MN deficiency predictor variables or features included in the models were child age in months, age of mothers, number of children under five, mother’s education, antenatal care (ANC) visit, postnatal care (PNC) visit, health check after delivery, place of delivery, current pregnancy status, currently breastfeeding, wealth index, region, place of residence, and media exposure (See details in Table 1). Moreover, the administrative region shapefiles were used to investigate the spatial variation in the prevalence of child MN deficiency.

**Table 1:** The description of the predictor variables considered in the analysis.

Variables	Descriptions
*Maternal level characteristics*	
Mother’s education	No education, primary, secondary, higher
Age of mothers	15–24, 25–34, 35–49 (Mothers current age)
Number of under five children	1, 2, 3 or more
*Community level characteristics*	
Place of residence	Urban/Rural
Media exposure	No/Yes
Wealth index	Poorest, poorer, middle, richer, richest
Region	Tigray, Afar, Amhara, Oromia, Somali, Benshangul, SNNPR, Gambela, Harari, Addis Ababa, Dire Dawa
*Obstetric characteristics*	
Antenatal care (ANC) visit	No visit, 1–3, >=4
Postnatal care (PNC) visit	No/Yes
Health check after delivery	No/Yes
Current pregnancy status	No or unsure/Yes
Place of delivery	Home/health facility
*Child level characteristics*	
Child age in months	6–8, 9–11,12–17, 18–23 (Child age in months)
Currently breastfeeding	No/Yes

#### 2.2.3 Feature selection

Feature selection is a critical step in predicting and interpreting high-dimensional datasets. We employed the Recursive Feature Elimination (RFE) method as a feature selection technique that uses a wrapper approach to select the most relevant features for a given ML model by recursively removing features from the dataset and training the model on the remaining features until the desired number of features is obtained (15). RFE is a valuable tool for identifying the most important features of MN deficiency in children and improving the predictive power of our ML models. Therefore, ML algorithms were applied to determine their predictive power and identify the most important determinants of child MN deficiency.

### 2.3 Machine learning methods

Machine Learning (ML) methods that were used in this study include SVM, LR, NN, RF, and NB. ML models have been used to rank relevant predictors of MN deficiency and to identify important predictors of health outcomes and other variables of interest.

We used the R programming language (version 4.2.2) and R packages sf (16), caret (17), and pROC (18) for data preprocessing and analysis. The performance of the ML algorithms was evaluated using metrics such as accuracy and the Area Under the Receiver Operating Characteristic curve (AUROC).

**Figure 1:**
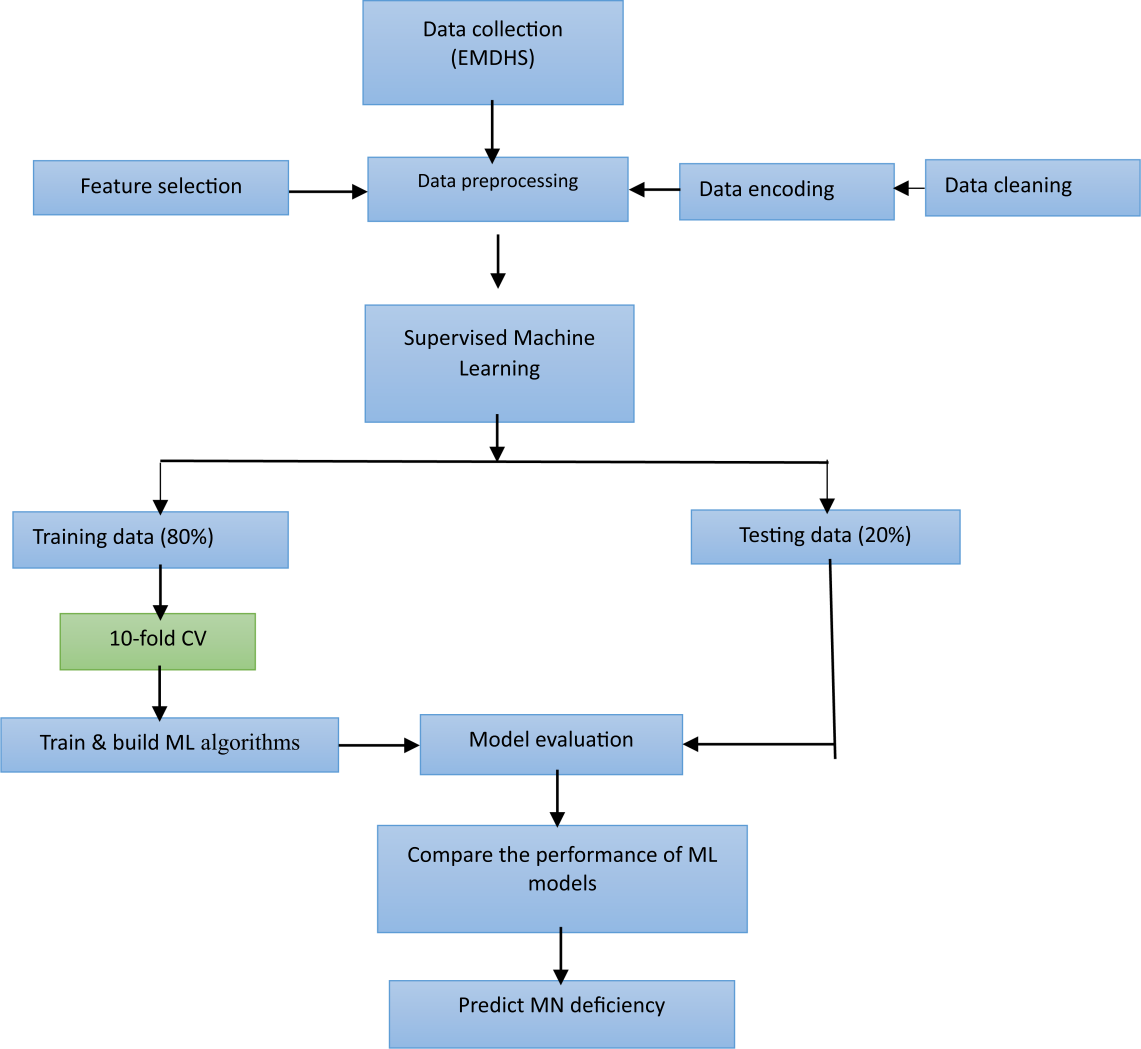
Flow chart of Machine learning approach.

In this study, we employed ML approaches by randomly dividing the dataset into two sets: 80% of it for the training set and 20% for the test set. The training set was used to train the model and the test set was used to evaluate the performance of the model. Standard ML accuracy measures were used to evaluate the prediction power of popular supervised ML algorithms, including SVM (13), LR (11, 14), NN (11–14, 19), RF (10–14, 20, 21), and NB (19). The ML algorithms were trained based on 10-fold cross-validation to optimize models. The overall pipeline of this study is shown in Figure 1. Figure 1 depicts the ML approach for predicting MN deficiency using EMDHS data. The approach involves several steps, including data collection, preprocessing, data cleaning and encoding, feature selection, building and evaluating ML algorithms, and comparing the performance of different models. The best-performing model was then used to predict MN deficiency. Following this approach, this study aimed to develop accurate and reliable predictive models that can inform public health policies and promote child development in Ethiopia.

Support Vector Machine (SVM) is a supervised ML model used for regression and classification that creates a hyperplane or set of hyperplanes in a high- or infinite-dimensional space. The objective is to maximize the margin between the nearest training points or support vectors of each class and the separating hyperplane. The best separation border is represented by the hyperplane with the largest available margin. To conduct linear separation, data must be transformed into higher dimensions using kernel functions. Non-linear classification tasks can be successfully completed using SVM, which is successful on complicated issues with little training data because of its generalization capabilities (22).

Logistic Regression (LR) is a statistical machine learning algorithm for binary classification problems that models the probability of an input data point belonging to a particular class. LR applies a logistic sigmoid function to the weighted sum of input predictors to estimate the probabilities, then thresholds the output to make a binary prediction. Moreover, it assumes a linear relationship between the log-odds of the outcome and the input predictors and can handle numerous predictor variables. It does not require linear relationships between dependent and independent variables, and penalization can control overfitting. The interpretability of model coefficients and probabilities makes logistic regression a popular starting classifier for machine learning applications involving binary prediction (23, 24).

The Random Forest (RF) is a popular algorithm for supervised ML that is used to solve classification and regression issues. It generates decision trees from randomly chosen data samples, gets predictions from each tree, and uses a majority vote to determine the optimal solution. RF also ranks the significance of each predictor using the mean decrease in accuracy (24–26).

The Neural Network (NN), also known as an Artificial Neural Network (ANN), is an ML model that uses a network of functions to recognize and translate a data input of one form into a desired output. The notion of NN was based on the biology of humans and how neurons work together in the human brain to understand information from the senses. NNs learn from labeled training data by adjusting the connection weights between layers of simple processing units, which enables them to model complex nonlinear relationships for applications in prediction, classification, and clustering (24, 27).

Naive Bayes (NB) is a supervised machine learning algorithm classifier based on Bayes’ theorem with independence assumptions between the features that simplifies the computation needed to estimate likelihood and posterior probability, making Naive Bayes a fast, scalable classifier that tends to perform very well on a variety of data despite its simplicity and restrictive assumptions (28).

### 2.4 Model performance evaluation

Different model performance metrics, including precision, recall or sensitivity, specificity, accuracy, F1 score, Receiver Operating Characteristics (ROC) curves, and ROC Area Under the Curve (ROC AUC) scores, were used to compare the performance of ML models or classifiers (24, 29).

A confusion matrix for binary classification is a two-by-two matrix that displays the values of True Positives (TP), False Negatives (FN), False Positives (FP), and True Negatives (TN) resulting from the predicted classes of data. By analyzing the confusion matrix, we can calculate various performance metrics such as recall (or sensitivity), specificity, and accuracy. The TP and TN represent correct classifications by the model, whereas FN and FP are incorrect predictions.

Recall (sensitivity) also called True Positive Rate (TPR) measures how many of the positive samples are captured by the positive predictions


$$TPR=\frac{TP}{\left(TP+FN\right)}$$

Specificity is another performance metric used in binary classification that measures the proportion of negative samples that are correctly identified by the model. Specifically, it measures the ability of the model to correctly predict negative samples as negative.


$$\mathrm{Specificity}=\frac{TN}{\left(TN+FP\right)}$$

Accuracy is a commonly used performance metric in binary classification that measures the proportion of samples that are correctly classified by the model out of all the samples it has predicted. It is calculated as:


$$\mathrm{Accuracy}=\frac{\left(TP+TN\right)}{\left(TP+FP+TN+FN\right)}$$

Precision also called positive predictive value (PPV) measures how many of the samples predicted as positive are actually positive.


$$\mathrm{Precision}=\frac{TP}{\left(TP+FP\right)}$$

The *F*_1_ score is the harmonic mean of precision and recall


F1=21Precision+1Recall=TPTP+FN+FP/2

The Receiver Operating Characteristic (ROC) curve is another standard tool used with binary classifiers, which plots sensitivity versus (1 − specificity). Measuring the Area Under the Curve (AUC) is one method of comparing classifiers. AUC provides an aggregated value that illustrates the likelihood that each ML algorithm will accurately classify a random sample. The better the classifier, the more closely the ROC curve will hug the top left corner (24, 30).

## 3 Results

### 3.1 Descriptive results

Data from 1,455 children aged 6 to 23 months were included in the analysis to assess the MN deficiency status in Ethiopia. Overall, 62.1% of them had not received any of the minimum recommended micronutrients and were therefore MN deficient. According to Table 2, the prevalence of MN deficiency was significantly higher among children whose mothers had no education (70.53%) compared to those with higher education (36.53%).

**Table 2:** Weighted prevalence and chi-square statistics of MN deficiency by demographic and other characteristics among children aged 6–23 months in Ethiopia (*n* = 1,455).

Predictors	Non-MN deficient (%)	MN deficient (%)	Chi square test statistic	*p* values
Region			126.37	0.000
Tigray	52.24	47.76		
Afar	16.63	83.37		
Amhara	29.53	70.47		
Oromia	42.07	57.93		
Somali	1.80	98.20		
Benishangul	47.52	52.48		
SNNPR	49.58	50.42		
Gambela	57.06	42.94		
Harari	45.81	54.19		
Addis Adaba	56.92	43.08		
Dire Dawa	43.18	56.82		
Place of residence			2.4024	0.121
Urban	47.81	52.19		
Rural	35.99	64.01		
Media exposure			7.8064	0.005
No	32.05	67.95		
Yes	52.57	47.43		
Number of under 5 children			12.199	0.002
1	40.19	59.81		
2	42.63	57.37		
3 or more	21.16	78.84		
Wealth index			28.89	0.000
Poorest	19.71	80.29		
Poorer	38.89	61.11		
Middle	38.47	61.53		
Richer	46.23	53.77		
Richest	52.88	47.12		
Current pregnant			1.053	0.305
No or unsure	39.79	60.21		
Yes	31.83	68.17		
Currently breastfeeding			0.298	0.5852
No	35.29	64.71		
Yes	40.03	59.97		
Maternal age			1.9302	0.381
15–24	38.67	61.33		
25–34	42.17	57.83		
35–49	32.75	67.25		
Maternal education level			23.465	0.000
No education	29.47	70.53		
Primary	44.84	55.16		
Secondary	48.20	51.80		
Higher	63.47	36.53		
ANC visit			8.49	0.014
No visit	26.51	73.49		
1–3 visits	39.30	60.70		
>=4 visits	46.15	53.85		
Place delivery			0.44	0.51
Home	36.17	63.83		
Health facility	41.75	58.25		
Health check after delivery			0.83	0.363
No	38.58	61.42		
Yes	45.94	54.06		
PNC check			0.52	0.47
No	38.52	61.48		
Yes	44.55	55.45		
Child age in months			13.46	0.003
6–8	28.07	71.93		
9–11	33.55	66.45		
12–17	36.88	63.12		
18–23	52.03	47.97		

MN, micronutrient; ANC, antenatal care; PNC, postnatal care; SNNPR, Southern Nations Nationalities and People Region.

The prevalence of MN deficiency decreases as the child’s age increases, with the lowest percentage of deficiency found in the 18–23 month age group (47.97%). MN deficiency is also significantly prevalent among children whose mothers have no media exposure (67.95%) compared to those with media exposure (47.43%). The results also suggest that as the wealth quintile increases, the prevalence of MN deficiency decreases, with the lowest percentage of deficiency found in the richest wealth quintile (47.12%) and the highest in the poorest (80.3%). The prevalence of MN deficiency also varies widely across regions, with the highest percentage of deficiency found in the Somali region (98.20%) and the lowest percentage of deficiency found in the Gambela region (42.94%) (Table 2).

According to Table 2, children whose mothers did not attend any ANC visits were more likely to have a MN deficiency (73.49%), compared to mothers who attended 1–3 ANC visits (60.70%) and those attended 4 or more visits (53.85%). Additionally, households with three or more children are more likely to experience a MN deficit (78.84%) than households with one or two children (59.81 and 57.3%, respectively).

### 3.2 Spatial distribution of childhood MN deficiency

**Figure 2:**
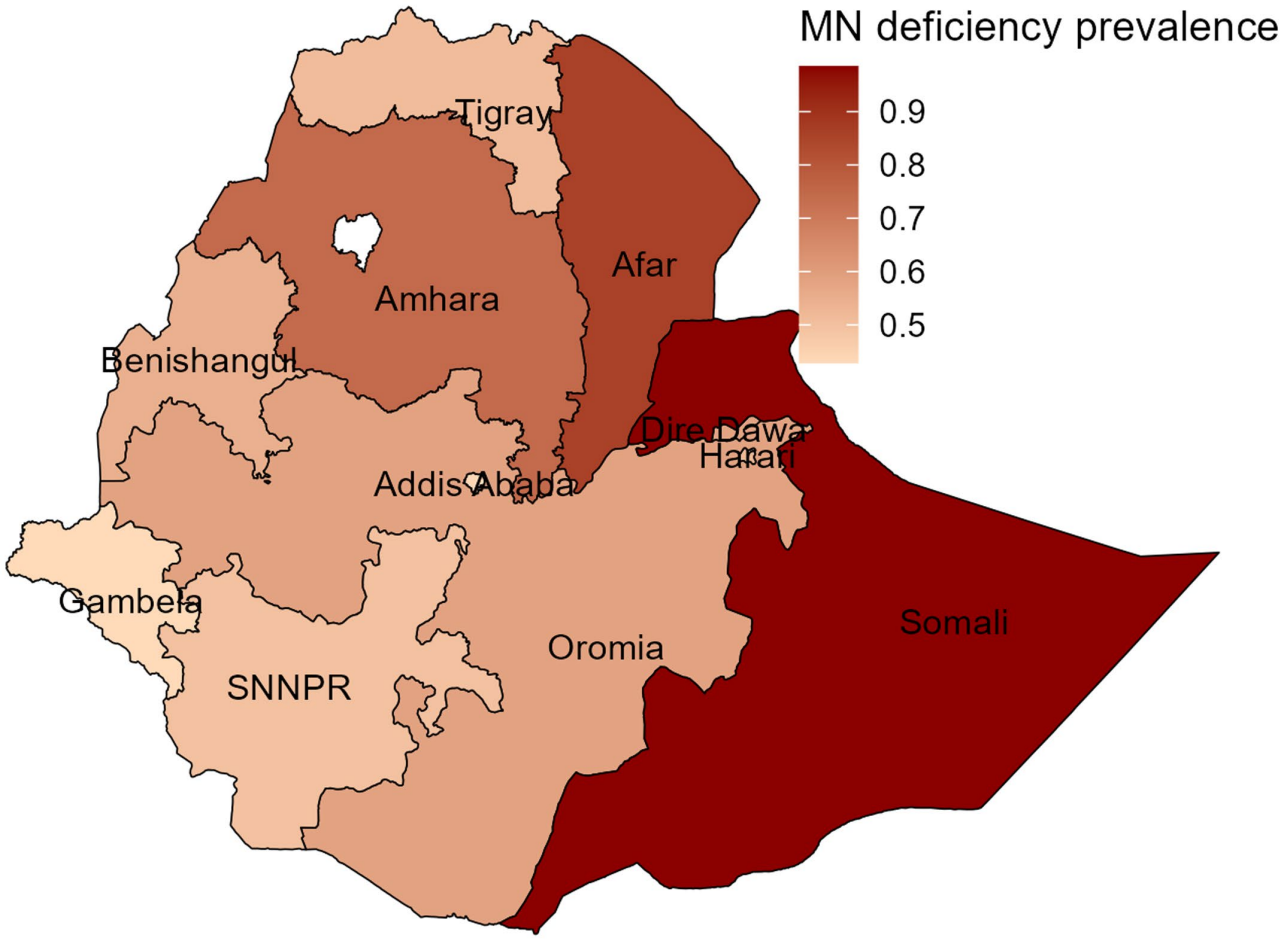
Spatial variations in MN deficiency by administrative regions in Ethiopia, EMDHS, 2019.

As per the findings presented in Figure 2, the spatial variation of childhood MN deficiency was most prevalent in Somali, Afar, and Amhara regions, while Gambela, Addis Ababa, and Southern Nations, Nationalities, and Peoples (SNNP) were the least affected regions. The findings suggest that the eastern part of Ethiopia, which includes the Somali and Afar regions, and the Amhara region were severely affected by MN deficiency.

### 3.3 Predictive algorithms for child micronutrient deficiency

**Figure 3:**
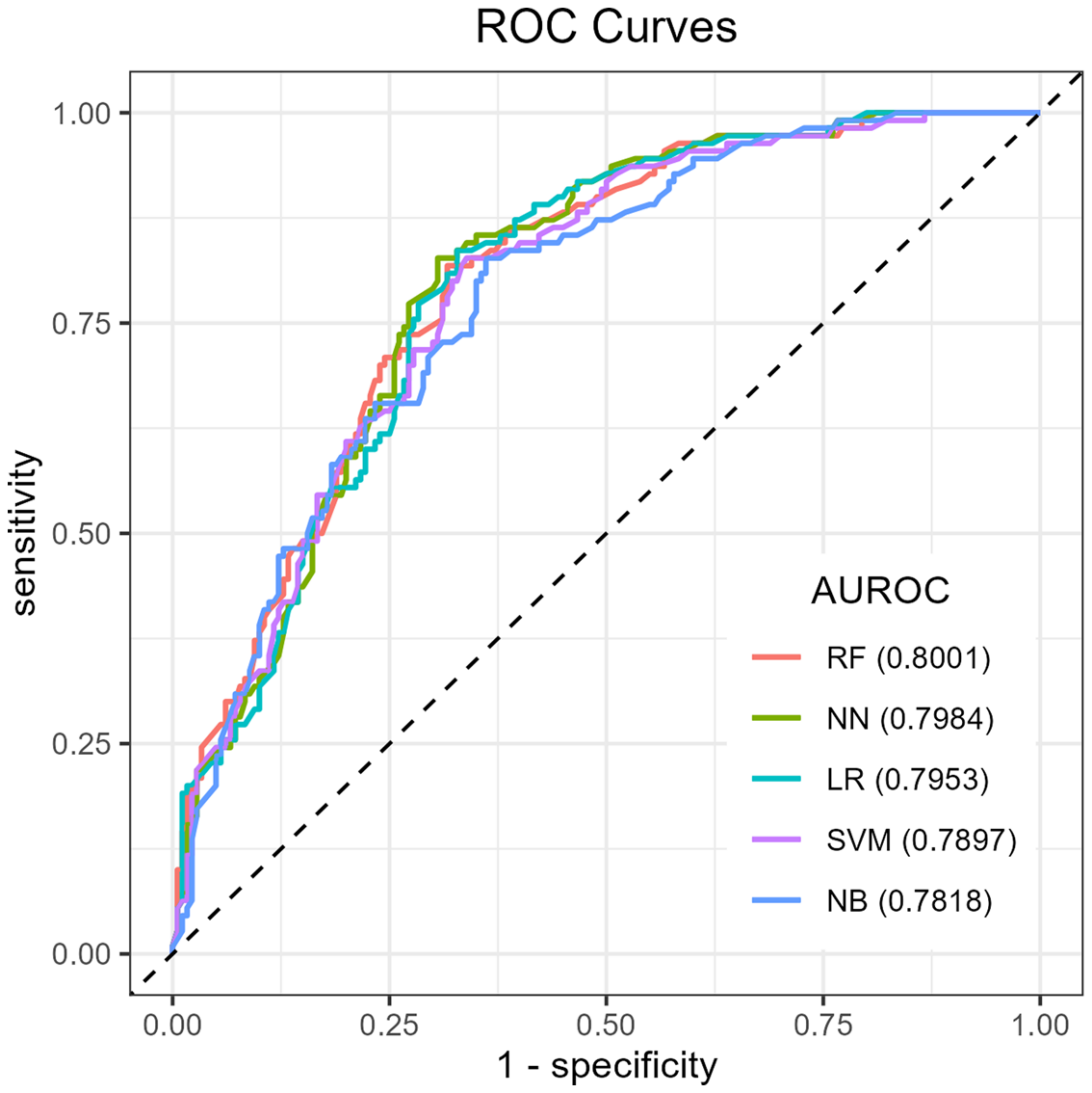
ROC curve for machine learning models in predicting childhood micronutrient deficiency. ROC, receiver operating characteristic; AUROC: area under the receiver operating characteristics.

The Recursive Feature Elimination (RFE) method was used to identify the features required to develop the ML algorithms on the training dataset. The results showed that RF had a relatively higher accuracy of 72.41% (95% CI: 66.89, 77.48), indicating its ability to correctly classify positive and negative cases. RF also achieved an AUROC of 80.01, suggesting good discriminative ability in distinguishing between positive and negative cases. The NPV of RF was found 69.23%, indicating its effectiveness in correctly identifying children without micronutrient deficiency. Additionally, the F1 score of RF was 79.59, indicating a balanced performance in terms of precision and recall, while NN had a slightly lower AUROC (79.84%) and accuracy (71.03%) compared to RF. Moreover, RF has the highest sensitivity (86.67%), meaning 86.67% of the children who are actually MN deficient are correctly identified by the model. In comparison to the other classifiers, Generalized Linear Model (GLM) had a slightly lower accuracy (70.69%) compared to RF, NN, and SVM and a relatively high AUROC score of 79.53% next to RF and NN. However, its sensitivity score of 80% was lower than those of RF and SVM. Finally, RF had the highest AUROC score (80.01%), whereas NB had the lowest (78.18%) (Figure 3). Based solely on the results presented in Table 3, RF, NN, and SVM were the top-performing algorithms, respectively, in terms of accuracy (Table 3). Thus, among all the algorithms utilized in our investigation, the RF algorithm performed the best in predicting the MN-deficient status of the cases, as evidenced by performance measures.

**Table 3:** Model evaluation metrics for all ML models as evaluated on the test data.

ML algorithms	Accuracy (95% CI) (%)	Sensitivity (recall) (%)	Specificity (%)	Precision (PPV) (%)	NPV (%)	F1-score (%)	AUROC (%)
SVM	71.03 (65.44, 76.19)	84.44	49.09	73.08	65.85	78.35	78.98
GLM	70.69 (65.09, 75.87)	80.00	55.45	74.61	62.89	77.21	79.53
RF	72.41 (66.89, 77.48)	86.67	55.45	73.58	69.23	79.59	80.01
NN	71.03 (65.44, 76.19)	80.00	56.36	75.00	63.27	77.42	79.84
NB	67.93 (62.22, 73.27)	57.78	84.55	85.95	55.03	69.10	78.18

ML, machine learning; GLM, generalized linear model; SVM, support vector machine; RF, random forest; NN, neural network; NB, Naive Bayes; PPV, positive predictive value; NPV, negative predictive value; AUROC, area under the receiver operating characteristic curve.

### 3.4 The important predictors of micronutrient deficiency

**Figure 4:**
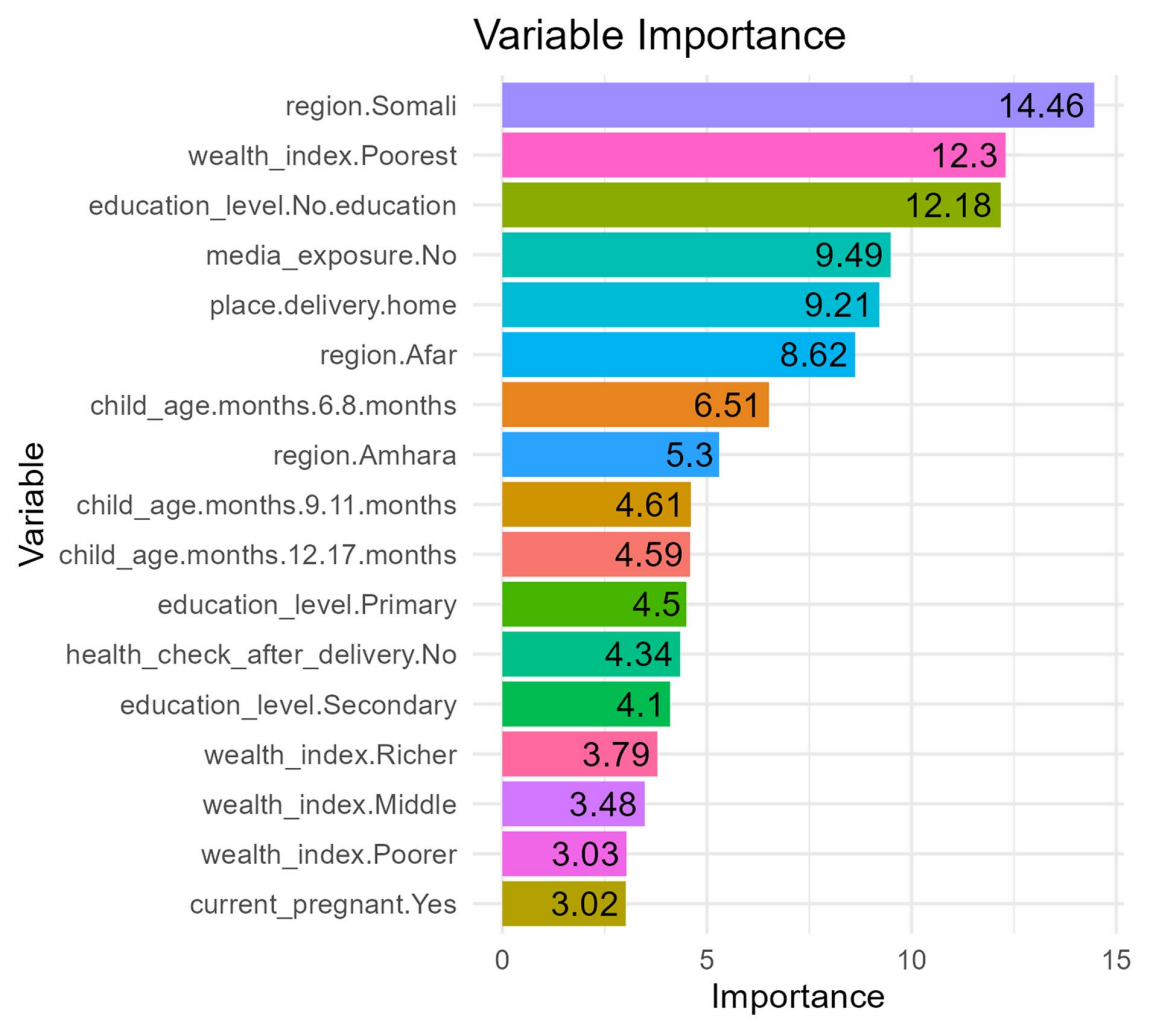
Variable importance from random forest.

The model evaluation findings, as discussed above, demonstrated that the random forest classifier was the best classifier in terms of accuracy and area under the receiver operating characteristics (AUROC) curve. Based on the most accurate classifier (RF), the top important predictors are presented according to their mean decrease accuracy (MDA) (Figure 4). Among the proposed predictors, the Somali region, the poorest wealth index, no maternal education, no media exposure, home delivery, the Afar region, and children aged 6–8 months were the top important predictors in their order of importance for MN deficiency among children aged 6–23 months in Ethiopia.

### 3.5 Spatial mapping of actual vs. predicted childhood MN deficiency prevalence

**Figure 5:**
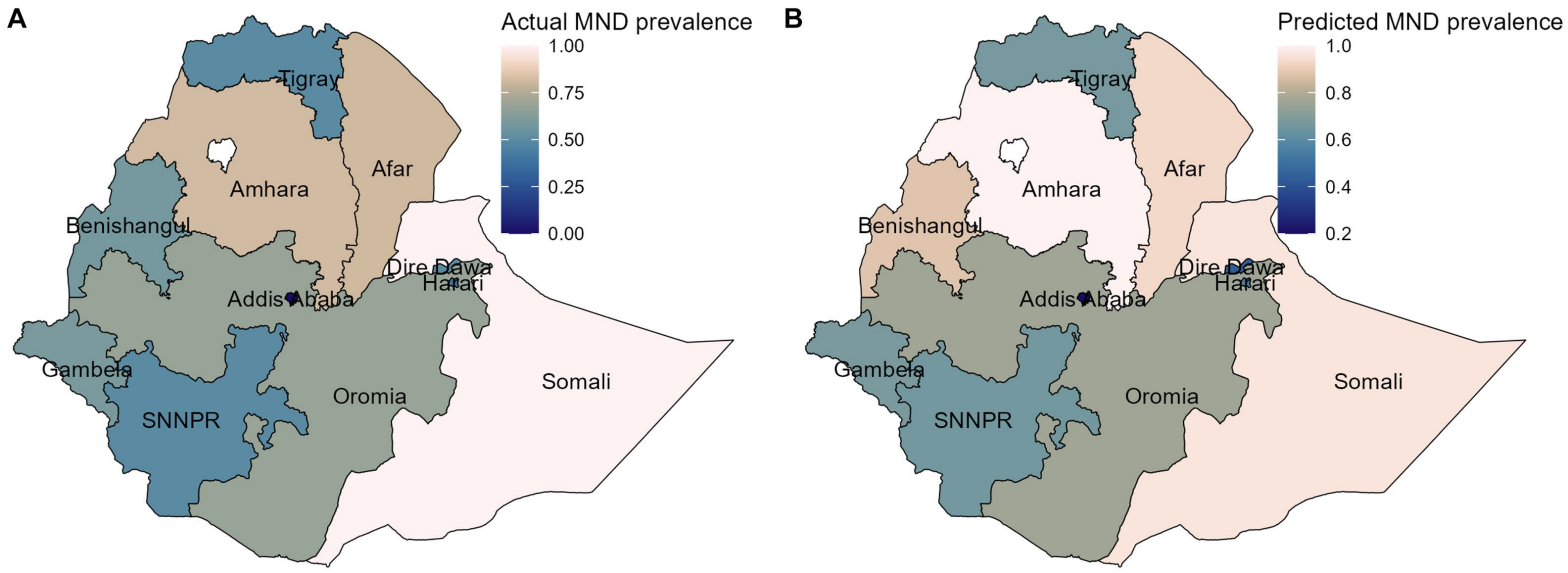
The spatial distribution of the actual **(A)**, and predicted **(B)** of MN deficiency prevalence on the test data. MND, micronutrient deficiency.

The spatial variation in Figures 5A,B depicts the actual and predicted prevalence of childhood MN deficiency for each region in the test data, respectively. To predict the regional prevalence of MN deficiency, our best predictive model (RF) was employed. Upon visual inspection of the map, we observed that while some discrepancies existed between a few regions, the overall patterns of the observed prevalence were consistent with the predicted prevalence of child MN deficiency. This suggests that our predictive model (RF) was reliable and can be used to predict the childhood MN deficiency prevalence in areas where data are lacking.

### 3.6 Classical logistic regression analysis

In contrast to the machine learning models, the traditional logistic regression model provides interpretable odds ratios for each predictor. Based on the results presented in Table 4, the region where the child lives, wealth index, maternal education level, and child age in months were found to be significant predictors of micronutrient deficiency among children aged 6–23 months in Ethiopia. Specifically, children living in the Somali and Afar region had 31.20 and 4.75 times higher odds of MN deficiency, respectively, compared to children in the SNNP region. Children in the poorest wealth index category had 4.75 times higher odds of micronutrient deficiency compared to children in the richest wealth index category. Moreover, the study found that a lower maternal education level and a younger child’s age were significantly associated with higher odds of micronutrient deficiency in children. Specifically, no education, primary, and secondary education in mothers were associated with 2.50, 1.96, and 1.91 times higher odds, respectively, compared to higher education. Children aged 6–11 months had 1.78 times higher odds of MN deficiency compared to those aged 18–23 months (Table 4).

**Table 4:** Logistic regression model results for factors associated with child MN deficiency (based on training data).

Characteristics	Adjusted odds ratio (AOR)	Confidence interval (CI)		*p* value
		Lower	Upper	
(Intercept)	0.13	0.06	0.29	0.000
*Region*				
Tigray	1.30	0.75	2.28	0.351
Afar	4.75	2.46	9.55	0.000
Amhara	2.53	1.48	4.36	0.001
Oromia	1.30	0.79	2.15	0.296
Somali	31.20	9.02	197.20	0.000
Benishangul	1.08	0.63	1.88	0.771
Gambela	0.58	0.32	1.04	0.070
Harari	1.66	0.91	3.04	0.100
Addis Adaba	1.94	0.99	3.81	0.053
Dire Dawa	1.78	0.96	3.34	0.071
*SNNP (Ref)*				
Media exposure				
No	1.39	1.00	1.95	0.053
*Yes (Ref)*				
Wealth index				
Poorest	1.81	1.09	3.01	0.021
Poorer	1.11	0.69	1.78	0.666
Middle	1.17	0.73	1.88	0.525
Richest	1.01	0.62	1.64	0.968
Richer (Ref)				
*Current pregnant*				
No (Ref)				
Yes	1.61	0.92	2.90	0.102
Education level				
No education	2.50	1.40	4.52	0.002
Primary	1.96	1.13	3.45	0.018
Secondary	1.91	1.02	3.62	0.045
Higher (Ref)				
Place of delivery				
Home	1.18	0.85	1.62	0.322
Health center (Ref)				
*Health check after delivery*				
No	1.37	0.91	2.05	0.133
Yes (Ref)				
Child age in months				
6–8	2.77	1.84	4.19	0.000
9–11	2.32	1.54	3.54	0.000
12–17	1.75	1.26	2.43	0.001
18–23 (Ref)				

AOR, adjusted odds ratio; CI, confidence interval; Ref, reference category.

## 4 Discussion

In this study, we found that children aged 6–23 months had a significant prevalence of MN deficiency, which accounted for 62.1% of children in Ethiopia. This finding highlights the highest MN deficiency compared with other studies conducted in East Africa (31), including Ethiopia (1). The difference in results can be explained by the influence of sample size because the current survey was a mini-demographic survey. Moreover, we found strong associations between certain demographic and socio-economic factors and the prevalence of micronutrient deficiency, such as poverty, lack of media exposure, young age, low maternal education, and larger household size. This finding is consistent with other studies in this area (1, 32, 33).

The findings of this study also showed considerable variations in MN deficiency among children across Ethiopian regions, as illustrated in the spatial map. MN deficiency is most prevalent in the eastern regions, such as Somalia and Afar, and in Amhara region, but least prevalent in the south-west, southern, and central regions in Gambella, SNNP, and Addis Ababa, respectively. Evidence of similar geographical variabilities in MN deficiency has been shown (1, 31, 34). These findings highlight the need for targeted interventions that address the specific needs of different population groups in the eastern regions of Ethiopia.

In terms of predictive ML algorithms, the random forest algorithm was found to have the highest accuracy and AUROC score for predicting micronutrient deficiency. However, it is worth noting that while the logistic regression algorithm (GLM) had slightly lower accuracy compared to other algorithms such as NN, RF, and SVM, its advantage lies in producing more interpretable results in terms of the predictors estimated in the algorithm. Numerous machine learning (ML) approaches have been applied to health issues, including nutritional status (11, 14, 21, 35), asthma risk prediction (20), and childhood anemia (9). These studies have demonstrated high-quality and valid predictions, highlighting the potential of the ML approach in predicting health outcomes. Findings from the RF classifier reveal that the Somali region, the poorest wealth index, children of mothers who have no education, children whose mothers have no media exposure, home delivery, the Afar region, and children aged 6–8 months were the top important variables in their order of importance for predicting MN deficiency among children aged 6–23 months in Ethiopia (1, 31, 32).

The findings of this study indicated that the poorest household wealth index was an important predictor of child MN deficiency. This aligns with evidence that poverty and the poorest wealth index status contribute to childhood MN deficiency (31, 33). Children from low-income households often have limited access to nutritious food, which can lead to deficiencies in essential micronutrients. The implications of these findings highlight the need for targeted interventions aimed at addressing MN deficiency in low-income households. Besides, this study finds that home delivery was a significant risk factor for micronutrient deficiency. This suggests that women who give birth at home may not receive the same level of support and education on proper nutrition and infant care that they would receive in a healthcare facility (36).

Likewise, the significance of a child’s age in predicting micronutrient deficiency has been well documented in the literature (1, 31, 33), which supports the results of this study. Additionally, it seems that children aged 6 to 11 months are more vulnerable to micronutrient deficiencies. These findings suggest that there is a strong association between child age and micronutrient deficiency, with younger children being at a higher risk of deficiency. This highlights the importance of early interventions to promote optimal nutrition and prevent micronutrient deficiency in infants and young children in Ethiopia.

Furthermore, the results indicate that a lack of maternal education increases the risk of childhood micronutrient deficiency. Conversely, children of educated women have significantly lower rates of micronutrient deficiency (31, 33). These findings have important implications for addressing child micronutrient deficiency and further emphasize the need to improve women’s education in developing countries to promote better outcomes for children’s micronutrient status. Moreover, the findings indicate that parents who lack media exposure are also important predictors of childhood micronutrient deficiency, which is consistent with previous research conducted in India (35). This indicates that parental access to media can play a significant role in promoting good nutritional outcomes for children.

Additionally, this study investigated the spatial variation of the actual and predicted prevalence of MN deficiency using RF model, which highlighted the overall patterns of the observed prevalence that were consistent with the predicted prevalence of MN deficiency in children. This suggests that our predictive model (RF) was reliable and can be used to predict the prevalence of childhood MN deficiency in areas where data is lacking.

Moreover, the findings from the best-performing ML model (RF) are largely consistent with the traditional logistic regression analysis. Both the eastern region where the child lives, the wealth index, maternal education level, and child age in months were found to be significant predictors of micronutrient deficiency among children aged 6–23 months in Ethiopia. However, home delivery and media exposure emerged as important predictors in the ML models but not in conventional logistic regression. This suggests that the ML models may reveal previously unknown insights beyond traditional logistic regression approaches. Specifically, ML models could identify new influential variables for policy decision making that are missed by standard statistical methods (37). While the core findings aligned, ML provided the additional benefit of highlighting novel and potentially crucial MN deficiency factors not captured by traditional logistic regression.

## 5 Conclusion

The aim of this study was to evaluate the effectiveness of various ML algorithms and identify the most accurate and efficient algorithm for predicting micronutrient deficiencies. Accuracy and AUROC were used to evaluate the predictive power of the ML algorithms. The random forest algorithm was identified as the best model, achieving an accuracy of 72.41% and an AUROC of 80.01% on the test data. Thus, the Somali region, the poorest wealth index, children of uneducated moms, children whose parents have no media exposure, home delivery, the Afar region, and children aged 6–8 months were found to be the most important predictors of child MN deficits in their order of importance. Furthermore, the findings demonstrated considerable regional variations in the frequency of child MN deficit, particularly in Ethiopia’s eastern region. Although the RF model and traditional logistic regression model displayed more similar important predictors, the RF model was able to discover some crucial predictors that the conventional logistic regression model had missed. As a result, our model may provide better policy suggestions for children with MN deficiency. These findings underscore the importance of socioeconomic and spatial factors in the incidence of micronutrient deficiencies among Ethiopian children. Addressing these issues may result in better health outcomes for children within an age category of 6–23 months. The regional variation in the prevalence of MN deficiency emphasizes the need for targeted interventions that account for differences in the prevalence and risk factors of micronutrient deficiencies across different regions in Ethiopia.

## Data availability statement

The datasets presented in this study can be found in online repositories. The names of the repository/repositories and accession number(s) can be found at: https://www.dhsprogram.com/Data/.

## Ethics statement

This research has obtained approval to access the Datasets. Subsequent to the submission and request of the study concept, consent was granted by the Data Archivist of The Demographic and Health Surveys (DHS) Program. All data used adhere to the ethical standards of research. Furthermore, the data was managed in accordance with the Helsinki Declaration of the World Medical Association.

## Author contributions

LGG: Conceptualization, Data curation, Formal analysis, Funding acquisition, Methodology, Investigation, Software, Validation, Visualization, Writing – original draft. EYD: Conceptualization, Data curation, Methodology, Validation, Visualization, Writing – review & editing. JAY: Conceptualization, Data curation, Formal analysis, Methodology, Validation, Visualization, Writing – review & editing.
